# Aging and Replicative Senescence Have Related Effects on Human Stem and Progenitor Cells

**DOI:** 10.1371/journal.pone.0005846

**Published:** 2009-06-09

**Authors:** Wolfgang Wagner, Simone Bork, Patrick Horn, Damir Krunic, Thomas Walenda, Anke Diehlmann, Vladimir Benes, Jonathon Blake, Franz-Xaver Huber, Volker Eckstein, Petra Boukamp, Anthony D. Ho

**Affiliations:** 1 Department of Medicine V, University of Heidelberg, Heidelberg, Germany; 2 Heidelberg Academy of Sciences and Humanities, Heidelberg, Germany; 3 Helmholtz Institute for Biomedical Engineering – Cell Biology, Aachen University Medical School, Aachen, Germany; 4 Division of Genetics of Skin Carcinogenesis, Deutsches Krebsforschungszentrum (DKFZ), Heidelberg, Germany; 5 Genomics Core Facility, European Molecular Biology Laboratory, Heidelberg, Germany; 6 Surgical Clinic, Division of Traumatology and Reconstructive Surgery, University of Heidelberg, Heidelberg, Germany; Istituto Dermopatico dell'Immacolata, Italy

## Abstract

The regenerative potential diminishes with age and this has been ascribed to functional impairments of adult stem cells. Cells in culture undergo senescence after a certain number of cell divisions whereby the cells enlarge and finally stop proliferation. This observation of replicative senescence has been extrapolated to somatic stem cells *in vivo* and might reflect the aging process of the whole organism. In this study we have analyzed the effect of aging on gene expression profiles of human mesenchymal stromal cells (MSC) and human hematopoietic progenitor cells (HPC). MSC were isolated from bone marrow of donors between 21 and 92 years old. 67 genes were age-induced and 60 were age-repressed. HPC were isolated from cord blood or from mobilized peripheral blood of donors between 27 and 73 years and 432 genes were age-induced and 495 were age-repressed. The overlap of age-associated differential gene expression in HPC and MSC was moderate. However, it was striking that several age-related gene expression changes in both MSC and HPC were also differentially expressed upon replicative senescence of MSC *in vitro*. Especially genes involved in genomic integrity and regulation of transcription were age-repressed. Although telomerase activity and telomere length varied in HPC particularly from older donors, an age-dependent decline was not significant arguing against telomere exhaustion as being causal for the aging phenotype. These studies have demonstrated that aging causes gene expression changes in human MSC and HPC that vary between the two different cell types. Changes upon aging of MSC and HPC are related to those of replicative senescence of MSC *in vitro* and this indicates that our stem and progenitor cells undergo a similar process also *in vivo*.

## Introduction

The regenerative potential of our body decreases upon aging. Regenerative tissues depend on specialized adult stem cells, thus aging in these tissues can be interpreted as signs of aging in somatic stem cells [1]. Adult stem cells are characterized by the dual function to differentiate into different cell lineages and to self-renew for maintenance of the stem cell pool. It is, however, still controversial if this self-renewal also includes juvenation or if adult stem cells are doomed to undergo aging upon each cell division. It is unclear if adult stem cells undergo functional and molecular changes, if their number decreases because of aging, or if aging is due to extrinsic environmental factors without any effect on the stem cell pool [2], [3].

Research on adult stem cells is limited by the available methods for their identification and purification. Hematopoietic stem cells (HSC) represent the so far best described type of adult stem cells. They give rise to all different blood cell lineages and have been used for more than 40 years in clinical therapy. Nevertheless, it is still not possible to isolate a homogeneous population of long-term reconstituting HSC or to expand these cells *in vitro*. However, they can be enriched within the subset of CD34^+^ hematopoietic progenitor cells (HPC).

Mesenchymal stromal cells (MSC) represent another multipotent cell population that, given the appropriate culture conditions, is able to differentiate into different mesodermal cell lineages including osteocytes, chondrocytes and adipocytes. In the literature they are commonly termed “mesenchymal stem cells” but also here reliable markers for the sub-fraction that meets the specific stem cell criteria are yet elusive and therefore they should rather be named “mesenchymal stromal cells” [4]. MSC are predominantly isolated by plastic adherent growth under specific culture conditions and therefore they are notoriously heterogeneous [5], [6]. Nevertheless, human MSC raise hope in regenerative medicine and their use is concurrently tested in a fast growing number of clinical trials (www.clinicaltrials.gov) [7].

Aging of cells during *in vitro* culture is dependent on the number of cell divisions [8]. Within about 20 to 50 population doublings, cells enlarge, become more granular, and slow down their proliferation rate. Ultimately they irrevocably stop cell division although they remain metabolically active and can be maintained in this state for years. This phenomenon of replicative senescence was already described over forty years ago by Leonard Hayflick [9], [10] and since then, it is debated if the so-called “Hayflick limit” reflects the aging process of the whole organism *in vivo*. All human somatic cells that can be grown successfully in culture undergo cellular senescence *in vitro* [11]. For MSC research this is of specific interest as various studies demonstrated functional implications of their differentiation potential by long-term culture [12]–[16].

We have previously demonstrated that replicative senescence of MSC is a continuous process starting at the first cell passage [16]. Various genes were differentially expressed and these changes were very consistent in different donor samples. In continuation of this research we have now compared primary human MSC and HPC derived from donors of different age groups. The aim of this study was to determine the molecular sequel of aging in human stem and progenitor cells and to analyze if there is a molecular relationship between aging and replicative senescence.

## Results

### MSC from young and elderly donors

**Figure 1:**
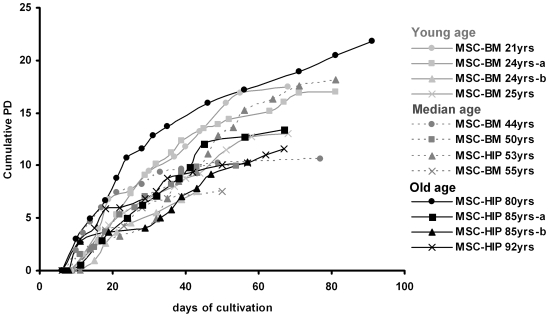
Long-term growth curves of mesenchymal stromal cells (MSC). Cells were isolated from human bone marrow from the iliac crest (BM) or from the femoral head (HIP) of donors that were either young (21–25 years), median aged (44–55 years) or old (80–92 years). Cell numbers were determined at the end of every passage and cumulative population doublings (PD) were calculated in relation to the cell numbers at the first passage.

In this study, we have isolated human MSC of young donors (21–25 years), median aged donors (44–55 years) and elderly donors (80–92 years; four donors per group). Plastic adherent colonies were observed in all donor samples after 7 to 10 days. Cell preparations fulfilled the criteria for definition of MSC [17]: 1) typical plastic adherent growth, 2) expression of CD13, CD29, CD44, CD73, CD90, CD105, CD146 and CD166 and absence of the surface molecules CD31, CD34 and CD45 (figure S1), and 3) *in vitro* differentiation potential towards osteogenic and adipogenic lineage (figure S2). We did not observe any difference in MSC of young and elderly donors with regard to growth morphology, immunophenotype and differentiation potential although there was some variation between different donor samples. Upon serial passaging, the cells underwent replicative senescence. This is reflected by the expression of senescence associated beta-galactosidase (SA-beta-gal; figure S3) and it was accompanied by decreased adipogenic differentiation potential and increased osteogenic potential as described before [16]. The proliferation rate decreased gradually until the cells finally stopped proliferation after 50 to 90 days (figure 1). The cumulative number of population doublings (PD) varied between 7 and 22 plus an estimated 7 to 9 population doublings during the initial colony formation. Thus, the total number of cumulative population doublings would be between 14 and 31 corresponding to a 10^4^-fold to 10^9^-fold expansion as also described by others [12], [18]–[20]. Long-term growth curves differed considerably between the different donor samples but there was no clear association between the maximal number of PD and donor age.

### Age-induced gene expression changes in MSC

**Figure 2:**
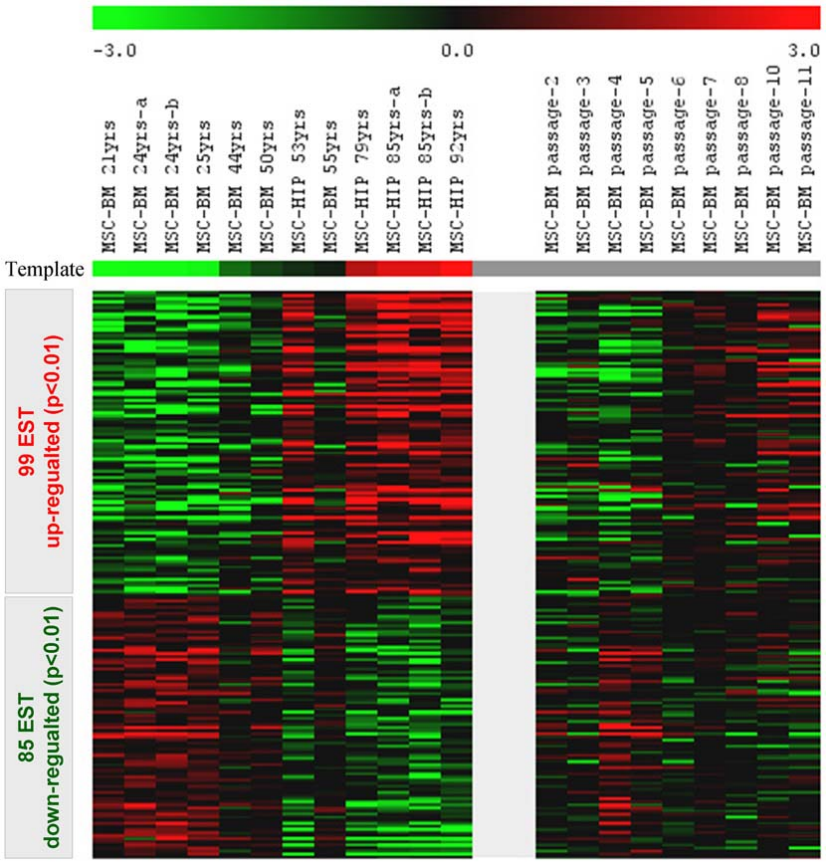
Age-induced gene expression in MSC is similar to replicative senescence. Global gene expression profiles of 12 MSC samples of different donor age were analyzed by Affymetrix technology. Statistical analysis by template matching (PTM) revealed that 99 ESTs were significantly up-regulated (red) and 85 ESTs were down-regulated (green) with increasing donor age. These differentially expressed genes were further analyzed within the dataset of nine different passages of the same MSC donor sample (44 years old). Colour coding in the heat map demonstrates that gene expression changes upon aging are also reflected by replicative senescence of MSC *in vitro*.

MSC from different-aged donors were harvested after the second passage to determine age-induced changes in their gene expression profiles. 99 expressed sequence tags (ESTs; including 67 non-redundant genes) were significantly up-regulated with increasing donor age whereas 85 ESTs (including 60 non-redundant genes) were significantly down-regulated (table S1, figure 2). Among the up-regulated genes were: mesenchyme homeobox 2 (MEOX2) that functions as a negative regulator of proliferation in several mesodermal tissues and that is most affected in fibroblasts of patients with Hutchinson-Gilford progeria syndrome [21]; short stature homeobox 2 (SHOX2) that is thought to be responsible for idiopathic short stature [22]; and HOXC6, which is part of a developmental regulatory system. On the other hand, various homeobox genes were age-repressed including HOXA5, HOXB3, HOXB7 as well as the paired-like homeodomain transcription factor 2 (PITX2).

**Figure 3:**
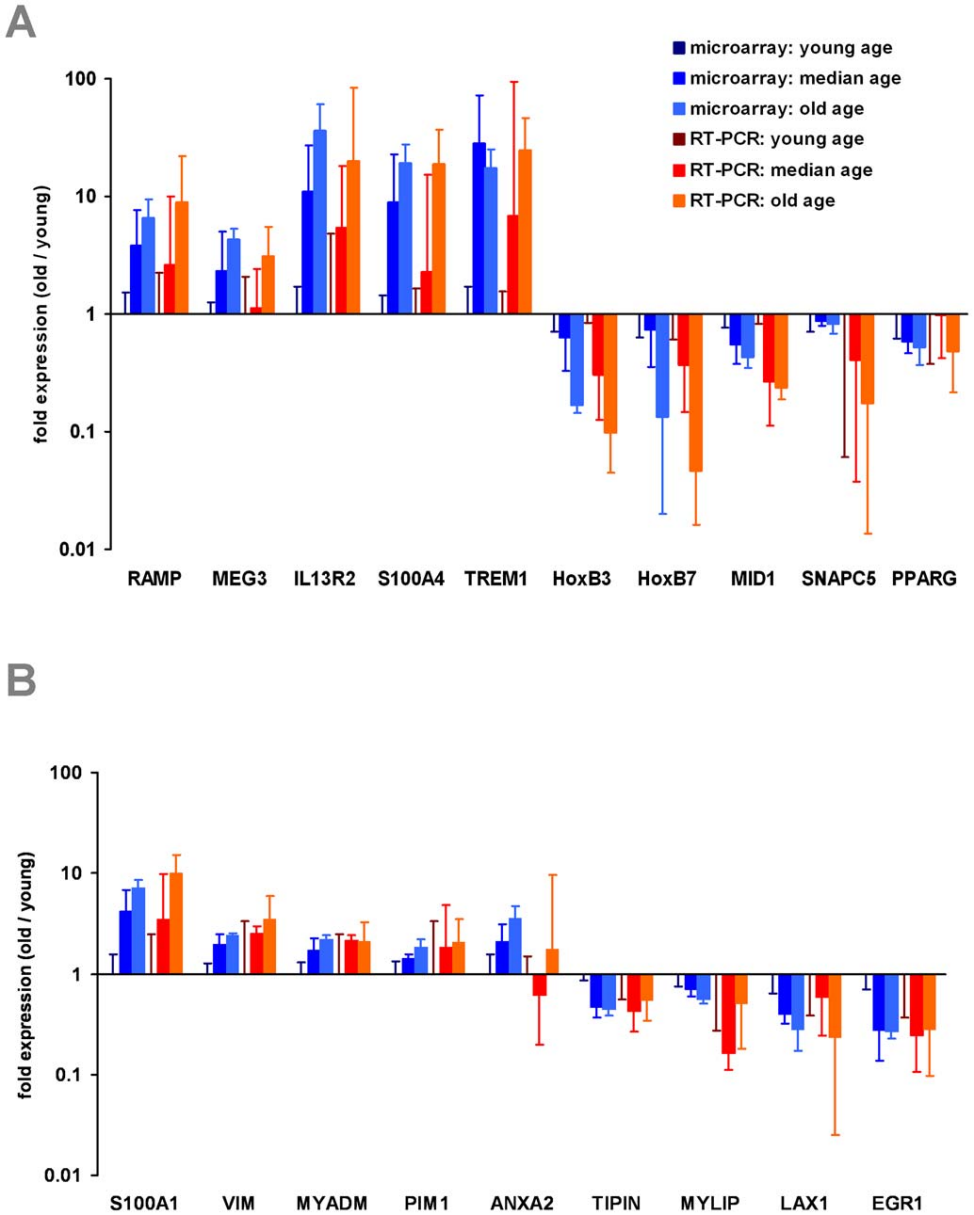
QRT-PCR validation of differential gene expression. Age associated gene expression changes in MSC were validated by quantitative RT-PCR. 10 genes were selected that have been differentially expressed in microarray data (A): regeneration associated muscle protease (RAMP); maternally expressed 3 (MEG3); interleukin 13 receptor, alpha 2 (IL13RA2); S100 calcium binding protein A4 (S100A4) and triggering receptor expressed on myeloid cells 1 (TREM1) were age-induced. Homeobox B3 (HOXB3) and Homeobox B7 (HOXB7); midline 1 (MID1); small nuclear RNA activating complex, polypeptide 5 (SNAPC5) and peroxisome proliferator-activated receptor gamma (PPARG) were age-repressed. Furthermore, we have validated age associated changes in HPC for 9 genes (B): S100 calcium binding protein A10 (S100A10); vimentin (VIM); myeloid-associated differentiation marker (MYADM); pim-1 oncogene (PIM1) and annexin A2 (ANXA2) were age-induced. Timeless interacting protein (TIPIN); myosin regulatory light chain interacting protein (MYLIP); lymphocyte transmembrane adaptor 1 (LAX1) and Early growth response 1 (ERG1) were age-repressed. Protocadherin 9 (PCDH9) was not amplified in HPC from elderly donors whereas interleukine 7 receptor (IL7R) was not amplified in young samples (not presented in the figure). Differential gene expression was always calculated in relation to the mean of young samples. The mean fold-ratio (±SD) is demonstrated for median aged and old donor samples. RT-PCR results (red) were always in line with microarray data (blue) for all genes tested.

To validate microarray data by independent means, we have performed quantitative RT-PCR. Five up-regulated and five down-regulated genes were selected for differential gene expression analysis upon aging of MSC. The results for the 12 MSC samples used for microarray analysis were in line with the microarray data demonstrating the reliability of the method (figure 3A). Furthermore, we have studied MSC from two young (26 and 29 years), two median aged (35 and 45 years) and two elderly (76 and 85 years) donors. Overall, age-associated differential gene expression could be verified for all genes tested (figure S4A).

### Aging of MSC *in vivo* correlates with senescence *in vitro*

We have previously demonstrated that replicative senescence of MSC is associated with continuous changes in their gene expression profiles [16]. For a direct comparison with age-induced gene expresssion changes we have reanalyzed the data of 9 different passages of the same donor (BM, 44 years old) by using the same statistical methods. 1257 ESTs (including 721 non-redundant genes) were significantly up-regulated in higher passages, whereas 698 (including 481 non-redundant genes) were down-regulated (figure S5; table S2). Subsequently, we have compared age-induced differential gene expression and replicative senescence associated gene expression changes. The color coded presentation of profiles revealed that many gene expression changes upon aging are also differentially expressed upon serial passaging *in vitro* (figure 2). Alternatively we have compared replicative senescence of different donor samples using the statistical methods as described in our previous work and again there was a correlation with differential expression upon aging. Thus, long-term culture of MSC induced continuous changes in the global gene expression profile and these changes were related to differential gene expression of MSC from different donor age.

### Gene expression changes in HPC upon aging

Furthermore, we have isolated the CD34^+^ fraction of hematopoietic progenitor cells of different-aged donors to determine the impact of aging on their gene expression profiles. HPC were isolated from 4 cord blood samples and from mobilized peripheral blood of 15 healthy donors between 27 and 73 years. There was no correlation between the number of mono-nucleated cells (MNC), the number of CD34^+^ cells or, the percentage of CD34^+^ cells with regard to donor age, suggesting that the number of mobilized HPC does not vary significantly upon aging (figure S6). Gene expression analysis revealed that 776 ESTs (including 432 non-redundant genes) were significantly up-regulated with increasing donor age whereas 704 ESTs (including 495 non-redundant genes) were down-regulated (table S3). Among the age-induced genes were: laminin A (LMNA) that is mutated in Hutchinson-Gilford progeria [21], [23]; the glycoprotein clusterin (CLU) that plays a role in apoptotic cell death and aggregates within plaques in the brains of Alzheimer's patients; CD44; annexins ANXA1, ANXA2 and ANXA3; the homeobox genes HOXA7, HOXB5, HOXB6 and HOXB7; and a remarkable over-representation of genes involved in major histocompatibility complexes (HLA-A, HLA-B, HLA-C, HLA-DRB1, HLA-DRB3, HLA-DMA, HLA-E and HLA-F) that has also been described for aging of murine leukocytes [24], [25]. In contrast, we did not observe an effect of aging on the expression of the CD34 gene in HPC.

To validate the microarray data we have selected six up-regulated and five down-regulated genes for RT-PCR analysis (figure 3B) and the results were further verified for eight independent samples (figure S4B).

### Aging of HPC *in vivo* correlates with replicative senescence of MSC

**Figure 4:**
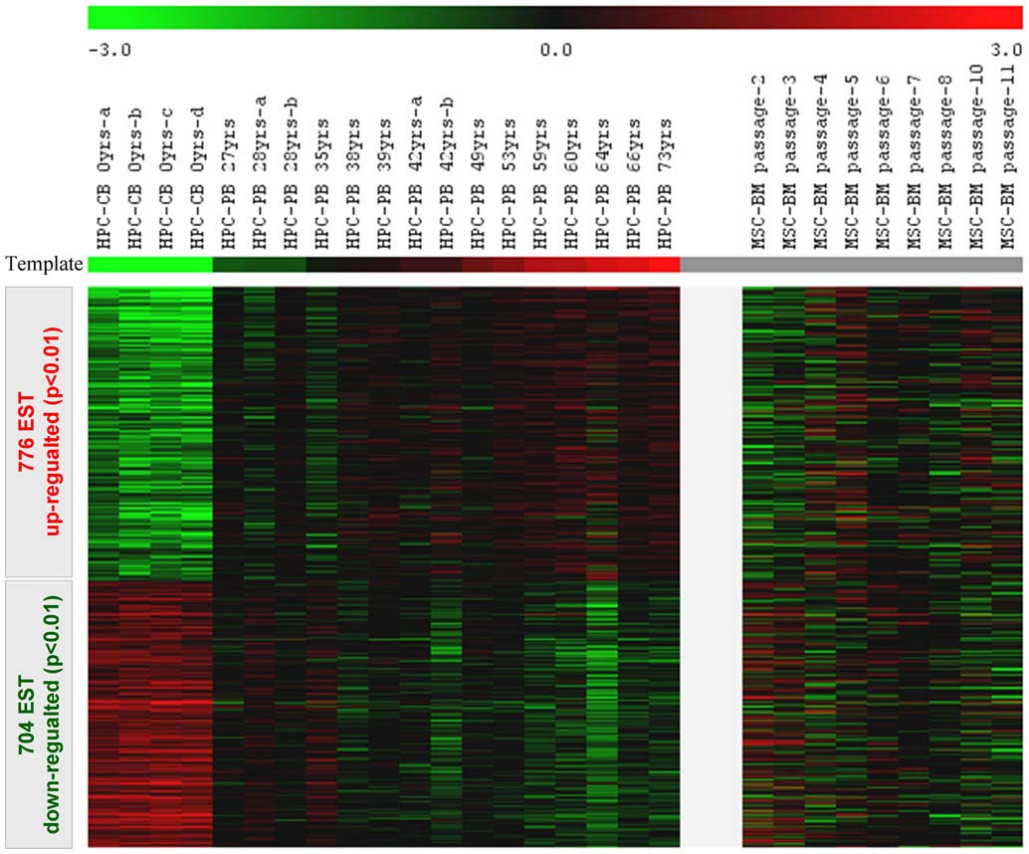
Age-induced gene expression of hematopoietic progenitor cells (HPC). CD34^+^ HPC were isolated from cord blood (CB) or from mobilized peripheral blood (PB) of healthy donors (27–73 years). Gene expression profiles revealed that 776 ESTs were significantly age-induced (red) and 704 ESTs were age-repressed (green). We have combined the age-induced gene expression of HPC with replicative senescence data in MSC. The heat map demonstrated here indicates an association between the two data sets.

Just as any somatic cell, also HPC can only be cultured *in vitro* for a limited number of cell divisions. Reliable methods for selective expansion of HPC are yet elusive and the progeny results predominantly in more differentiated cell types. This is reflected by the rapid decline of CD34 expression after only a few cell divisions [26]. Therefore, gene expression changes upon long-term culture of HPC might rather be due to differentiation than to replicative senescence. Instead, we have compared age-induced gene expression changes in HPC with the previously described dataset on replicative senescence in MSC. Color coding in the heat map demonstrated that age-induced gene expression changes in HPC were related to gene expression changes upon replicative senescence of MSC *in vitro* (figure 4).

### Age associated gene expression changes vary in different cell types

**Figure 5:**
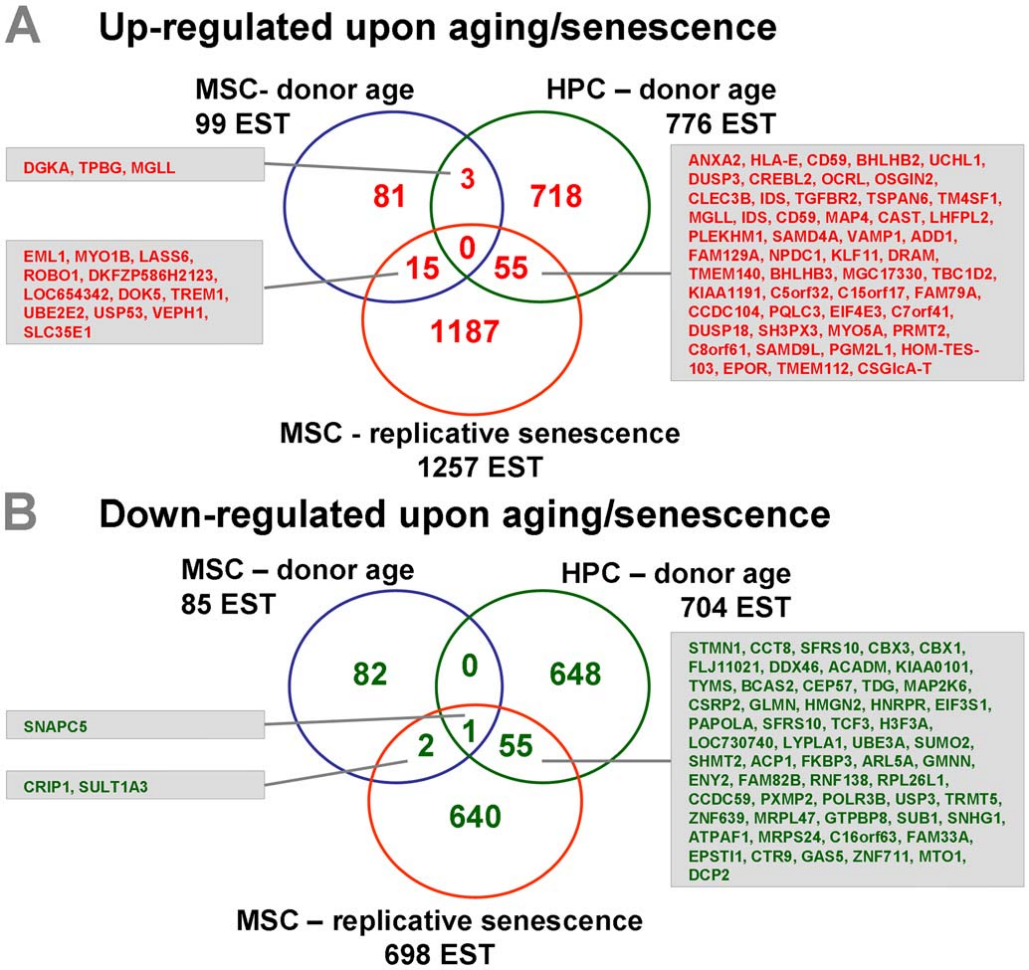
Combination of the three datasets. Differential gene expression of the three datasets (*MSC–donor age; HPC–donor age* and *MSC–replicative senscence*) was further compared. Venn diagrams demonstrate the overlap of differentially expressed ESTs that are up-regulated upon aging (A) or down-regulated (B). Differentially expressed genes are indicated as short cut (Hugo name). There was only little overlap between *MSC–donor age* and *HPC–donor age*. However, both datasets demonstrated a relatively high overlap with the dataset *MSC–replicative senescence*.

The three datasets (*MSC-donor age*; *MSC-replicative senescence* and *HPC-donor age*; (figure 5) were then collectively analyzed. Overall, there was little overlap and only the small nuclear RNA activating complex 5 (SNAPC5) was consistently down-regulated upon aging in MSC and HPC as well as during replicative senescence of MSC [27]. Notably, age-induced gene expression in MSC and HPC showed only moderate overlap indicating that age-associated changes differ considerably between different cell types. However, there was a significant concordance of genes that were regulated upon aging of MSC and upon replicative senescence of MSC (P = 0.003). Other genes also revealed a concordance between aging of HPC and replicative senescence of MSC (P = 4.5×10^−8^). Thus, aging of human stem and progenitor cells *in vivo* and cellular senescence *in vitro* seems to be related to a comparable molecular basis.

Differential gene expression was further analyzed with regard to Gene Ontology (GO) categories. Several functional categories were significantly over-represented in each of the three datasets within either age-induced or age-repressed genes (figure S7). Similar functional categories were age repressed: GO categories related to the DNA repair, mitosis, transcriptional regulation and nucleus were age-repressed in MSC, HPC as well as in replicative senescence of MSC. In contrast, genes involved in categories for differentiation, plasma membrane and extracellular matrix were age-induced in MSC, HPC and replicative senescence.

Chromatin remodeling or confined epigenetic modifications might result in chromosomal hot-spots for age-induced gene expression changes. To test this hypothesis we have checked the representation of the differentially expressed genes within chromosomal regions (G-banding). Indeed, there were significant over-representations at specific chromosomal sites (table S4). However, these hot-spots did not coincide in up- and down-regulated genes of all three datasets (*MSC-donor age*; *MSC-replicative senescence* and *HPC-donor age*). This indicates that the underlying mechanism of aging and replicative senescence of adult stem cells is not attributed to regulation of gene expression at specific chromosomal locations.

### Analysis of telomerase activity and telomere length in HPC upon aging

**Figure 6:**
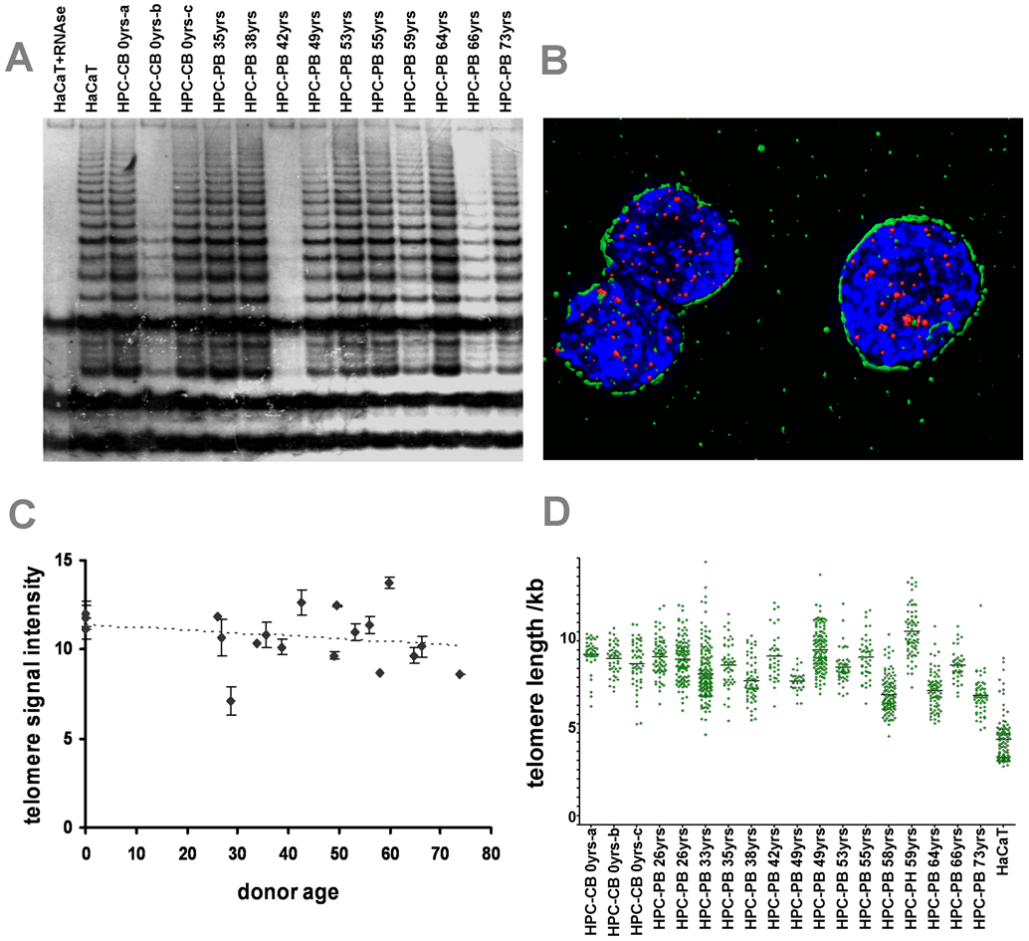
Telomerase activity and 3D reconstruction of telomeres in HPC upon aging. TRAP assay was performed on CD34^+^ cells of different donor ages. HaCaT cells exhibiting a similar telomerase activity as HeLa cells were used as a positive control [75]. Telomerase activity in HPC varied between hardly detectable and as high as in HaCaT cells with no clear association between the level of telomerase activity and donor age (A; HaCaT cell lysat = positive control, HaCaT lysat + RNAse = negative control). Telomere length in HPC was estimated using 3D image reconstruction. Cells were immunostained for CD34 (green), hybridized for telomeres with the telomere-specific PNA probe (red) and DNA counterstained with DAPI (blue) (B). Graphic presentation of the telomere signal intensity measurement (arbitrary units) of CD34^+^ cells from 19 different age donors demonstrates a weak tendency of telomere shortening during age (C; the slope of linear regression is not significant, P = 0.1034). The distribution of telomere length in individual cells revealed a tendency for higher variation in telomere length in HPC of elderly donors (D; P = 0.0450).

Although it is supposed that HSC are telomerase-positive and thus should maintain a stable telomere length [28], there is emerging evidence that telomere dysfunction due to telomere shortening can limit the maintenance and function of adult stem cells [29]. Thus, age-dependent telomere shortening may be causally associated with the age-dependent changes described above. To address this we additionally investigated the CD34^+^ HPC for their telomerase activity and telomere lengths. Using the TRAP (telomere repeat amplification protocol) assay to measure telomerase activity, we found all 13 HPC samples to be telomerase-positive. Also in line with previous reports, the activity varied from very weak levels to levels comparable to that seen in the immortal HaCaT skin keratinocytes, i.e. a level sufficient to maintain stable telomere length during serial passaging (figure 6) [30], [31]. Because of this variation, which was further confirmed by serial dilution analysis of all samples in order to avoid false negative (inhibitors) or positive (primer dimers) results (figure S8), an age-dependent decline was not obvious.

In addition, we also determined telomere length from all 19 HSC populations. Due to the low number of cells available from each isolation, we performed 3D Telo-FISF analysis, allowing for quantitation of telomere length of each individual cell [30]. This study showed an only weak tendency of age-dependent telomere shortening and no sign of subpopulations with increased telomere attrition. With the exception of one sample (mostly two apoptotic cells) we also did not detect up-regulation of p53 or the formation of p53BP1-positive damage foci thus excluding uncapped telomeres in the HSC populations including those from the oldest donors (figure 6). However, there was a tendency for an increasing interpersonal heterogeneity of telomere length with age. Since this heterogeneity did not simply reflect the respective level of telomerase activity these data may argue for a more complex regulation of telomerase and telomere length in HSC.

## Discussion

The deterioration of the regenerative potential upon aging might be due to functional changes in adult stem cells. To test this hypothesis we have investigated differential gene expression in primary, human MSC and HPC derived from different age groups. In this study, we demonstrate for the first time age-related gene expression changes in human MSC and HPC and that there is a moderate but significant concordance in the expression profiles upon aging *in vivo* and replicative senescence *in vitro*. It needs to be pointed out, that chronological age and biological age do not necessarily coincide. Multiparametric assessment of biological age might be valuable in this context. Furthermore, MSC and HPC preparations are heterogeneous and it is conceivable that they represent a mixture of different aged or senescent subsets. Further research will be necessary to address age-related changes on a single cell level to investigate the heterogeneity of aging within cell populations.

Expression of many homeobox transcription factors was regulated upon aging and most of these were age-repressed in MSC (including HOXA5, HOXB3 and HOXB7). On the other hand, several of these were age-induced in HPC (HOXA7, HOXB5, HOXB6 and HOXB7). Thus, homeobox transcription factors may not only function in morphogenesis and differentiation but they might also be involved in the process of aging. Their expression might be co-regulated for example by polycomb group genes such as EZH2 [32]. It is remarkable that there was only little overlap in age associated differential gene expression in MSC and HPC. Furthermore, we did not detect any chromosomal locations where age-induced gene expression changes are coherently over-represented upon aging of MSC and HPC. It needs to be taken into account that gene expression profiles reflect the effects of aging rather than the initiating mechanism. Hence, it might not be surprising that the molecular sequel of aging varies between different cell types with different regulatory networks, different environments and different cellular fates.

So far, only few studies have analyzed global gene expression patterns in somatic stem cells upon aging and these were performed in murine HPC [33]–[35]. Rossi and co-workers analyzed differential expression of HPC (c-kit^pos^, lineage^neg^, Sca-1^pos^, flk2^−^, CD34^−^) from young (2–3 month) and old (22–24 month) mice by microarray [34]. Chambers et al. have also isolated HPC (c-kit^pos^, lineage^neg^, Sca-1^pos^) from mice of different ages and analyzed differential gene expression [33]. Overall there was only little overlap between these two studies, as well as between these and our results. This could be due to differences between mice and men as well as to different cell fractions used in these studies. However, with regard to Gene Ontology categories there is some concordance between their and our results: age-repressed genes were over-represented in categories involved in genomic integrity, regulation of transcription and chromatin remodeling [33], [34]. Loss of these functions might underlie the process of aging.

Fundamentally different ways have been proposed to explain the process of aging: it might be evoked by stochastic or random, accidental events that accumulate throughout life until the cell looses its integrity or aging might be the result of a purposeful program driven by regulated changes in gene expression or epigenetic modifications [36]. The “disposable soma theory” suggests that the investments in durability and maintenance of somatic tissues are conformed to life expectancy [37], [38]. This concept is compatible with our observation that genes involved in genomic integrity and regulation of transcription are age-repressed. There is experimental evidence that aging involves DNA damage, accumulation of the cyclin-dependent kinase inhibitor p16INK4a, the p53 pathway and oxidative stress and their role in aging has been discussed [1], [39]–[42]. Epigenetic modifications such as DNA methylation or histone acetylation might result in age associated molecular changes [33], [43] and it is conceivable that such epigenetic changes repress pathways involved in genomic integrity, maintenance and repair.

The major question is whether replicative senescence does play a role in human aging. Several studies have shown an inverse relationship between donor age and the replicative life span *in vitro* for fibroblasts or MSC [13], [44], [45]. This effect is usually relatively small with a high variation between different donor samples [12], [46]. At least some of the variability was attributed to differences in donor health status, conditions for the biopsy and the initial CFU-F frequency in the bone marrow sample [47]. Furthermore, the pace of senescence might be affected by the culture conditions [19], [48]. In MSC preparations used in this study we did not discern any age-associated effects on replicative senescence. If the number of cumulative population doublings was not significantly affected by aging it is all the more surprising, that there was a significant association between age-induced gene expression changes and replicative senescence. These results indicate that the molecular sequels of aging *in vivo* and replicative senescence *in vitro* are based on similar mechanisms.

Progressive shortening of the telomeres or modified telomeric structures have been discussed to be the main trigger for replicative senescence and it has been anticipated that telomere shortening provides an internal clock. With every cell division the number of telomere repeats decreases and this has also been demonstrated for MSC [12], [14]. The process is counteracted by expression of telomerase in somatic stem cells [49], [50]. This is in line with our results where telomerase activity was detected in HPC. Vaziri et al. have demonstrated that CD34^+^CD38^−^ HPC from human bone marrow have shorter telomeres than those from fetal liver or cord blood [51]. In this study telomere length decreased only slightly upon aging and we did not detect subpopulations with very short telomeres or signs of telomere dysfunction (uncapped telomeres) in any of the samples. Hence, it is unlikely that age-induced gene expression changes in HPC are only due to telomere loss. Though discussed controversially, our data rather support studies by others that telomere shortening may not be the only reason for replicative senescence in hematopoietic cells *in vivo* [40], [52]–[54].

There is emerging evidence that aging is not purely a cell intrinsic process, but rather regulated by interaction with the cellular microenvironment. For example, Ju and co-workers have demonstrated that telomere dysfunction induces alterations in the microenvironment that affect aging of the hematopoietic system [55]. In general, adult stem cells have a slow turnover and reside in specialized niches, protected from the environment and only a few are activated at a time [33], [56]. By keeping adult stem cells in a quiescent state, the stem cell niche might also play a crucial role in regulating replicative senescence. Strong experimental data for this hypothesis derives form serial transplantation experiments of HSC in mice. The reconstituting ability declines continuously within 4 to 5 transfers [57], [58] and this decline is thought to be telomere-independent [59], although it has been reported that telomere length decreases by serial transplantation [60]. Recently, Wilson and co-workers have demonstrated that there is a dormant-fraction of HSC that divides only five times during the lifetime of mice and especially these dormant HSC posses repopulating activity upon serial transplantation [61]. The stem cell niche could therefore play a central role in maintaining a dormant pool of HSC to prevent replicative senescence over the lifetime of the organism [62].

In conclusion, the gene expression profiles indicate that our stem and progenitor cells are not protected from aging. Further research will be necessary to determine if aging also affects the differentiation potential of stem cells. For example the myeloid skewing of hematopoietic differentiation with age has been suggested to be due to intrinsic changes in HSC [63], [64]. If replicative senescence is involved in aging, we should either be running out of cell divisions or the increased time for cell division or impairments of differentiation compromise regeneration of organs, leading to their loss of function. Our data demonstrate that gene expression changes upon *in vivo* aging and *in vitro* replicative senescence are related.

## Materials and Methods

### Isolation of MSC from human bone marrow

MSC from human bone marrow were either isolated from bone marrow aspirates from healthy donors for allogeneic transplantation (BM) or from the caput femoris from elderly patients undergoing femoral head prosthesis (HIP) [65]. All samples were taken after written consent using guidelines approved by the Ethic Committee on the Use of Human Subjects at the University of Heidelberg. 20 ml bone marrow aspirate with heparin were taken from healthy donors for allogeneic bone marrow transplantation and immediately processed by ficoll density fractionation for isolation of mononuclear cells (MNC). Alternatively, the femur head was flushed with PBS under sterile conditions immediately after operation and subsequently the MNC were isolated by ficoll density fractionation. All samples were cultured under the same standardized culture conditions as described before [16], [66]–[68]. Tissue culture flasks were coated with 10 ng/ml fibronectin (Sigma) before use.

### Expansion and sampling of MSC

MSC were cultured at 37°C in a humidified atmosphere containing 5% carbon dioxide with medium changes twice a week. After 7–10 days, initial colonies were trypsinized and re-plated in a new culture flask (passage 1, P1). Upon sub-confluent growth (70%), cells were re-plated at a density of 10^4^ cells/cm^2^. Photo documentation and cell counting by using a Neubauer counting chamber was performed at every passage. Cumulative population doublings were calculated as previously described [46]. As cell numbers were first determined at P1, the cumulative doubling number was first calculated for P2. From P2 onward, there were enough cells for simultaneous expansion of one fraction and harvesting another fraction for subsequent analyses: 10^6^ cells were lysed in TRIzol and stored at −80°C for RNA isolation and 10^6^ cells were cryopreserved for immunophenotyping and *in vitro* differentiation.

### Immunophenotypic analysis and *in vitro* differentiation of MSC

Immunophenotypic characterization of MSC preparations was performed on a three-color FACScan (Becton Dickinson [BD], San Jose, USA) with a five-color upgrade (Cytek Dev. Inc., Fremont, Ca, USA) using the commonly used panel of antibodies as described before [16], [66]: CD13-allophycocyanin (APC, clone WM15, BD), CD29-fluorescein isothiocyanate (FITC, MEM-101a, abcam, Cambridge, UK), CD31-FITC (BD), CD34-phycoerythrin (PE, 8G12, BD), CD44-PE (g44-26, BD), CD45-FITC (2D1, BD), CD73-PE (AD2, BD), CD90-PE (G7, BD), CD105-PE (MHCD10504, BD), CD146-PE (P1H12, BD), CD166-PE (3A6, BD), CD184-PE (12G5, BD). To induce osteogenic differentiation, cells were re-plated at 2×10^4^ cells/cm^2^ and cultured for three weeks in DMEM with 10% FCS (Invitrogen), 10 mM β-glycerophosphate, 10^−7^ M dexamethasone, and 0.2 mM ascorbic acid and with medium changes every 3 to 4 days as previously described [68], [69]. After 21 days, cells were analyzed by Alizarin red staining. For adipogenic differentiation, cells were plated at 2×10^4^ cells/cm^2^ and cultured in DMEM with 10% FCS, 0.5 mM isobutyl-methylxanthine (IBMX), 1 µM dexamethasone, 10 µM insulin, 200 µM indomethacin, and Oil Red-O staining was performed after 21 days [70].

### Senescence associated beta-galactosidase staining

Expression of pH-dependent senescence associated beta-galactosidase (SA-beta-gal) activity was analyzed simultaneously in different passages of MSC of young and old donors using the SA-beta-gal staining kit (Cell Signaling Technology, Boston, MA).

### Isolation and sampling of human HPC

Human HPC were isolated within the CD34^+^ cell fraction of fresh umbilical cord blood (CB). Alternatively they were isolated form peripheral blood (PB) of healthy donors upon mobilization with granulocyte-colony stimulating factor (G-CSF) before leukapheresis procedure for allogeneic stem cell transplantation. All samples were taken after written consent using guidelines approved by the Ethic Committee on the Use of Human Subjects at the University of Heidelberg. Mononuclear cells were isolated after centrifugation on Ficoll-hypaque (Biochrom KG, Berlin, Germany). CD34^+^ cells were enriched using a monoclonal anti-CD34 antibody labeled with magnetic beads on an autoMACS system (Miltenyi Biotec, Bergisch-Gladbach, Germany). After additional staining with anti-CD34-APC (BD) further purification was achieved using the FACS-Vantage-SE flow cytometry cell sorter. Staining with propidium iodide (PI) was performed to allow exclusion of non-viable cells. Reanalysis revealed purity higher than 95%. 10^5^ cells were lysed in TRIzol and stored at −80°C for RNA isolation. From the same population cells were lysed in CHAPS buffer for TRAP assay and were transferred (cytospin centrifugation) to glass slides for telomere length analysis, respectively.

### RNA isolation

Total RNA was isolated using TRIzol reagent (Invitrogen, Paisley, Scotland) according to the manufacturer's instructions. RNA quality was controlled using the RNA 6000 Pico LabChip kit (Agilent, Waldbronn, Germany) and quantified with a NanoDrop ND-1000 Spectrophotometer (Nanodrop Technologies, Wilmington, USA).

### Microarray experiments

In this study we have compared three different sets of microarray data:

1. *MSC-donor age*: MSC samples were taken from 4 young donors (21–25 years), 4 median aged donors (44–55 years) and 4 old donors (80–92 years). For this comparison MSC were harvested after the second passage (P2). Two µg of total RNA were amplified with GeneChip one-cycle target labeling kit (Affymetrix, High Wycombe, United Kingdom) according to the manufacturer's instructions. Quality of amplified RNA was controlled by LabChip technology. GeneChip Human Genome U133_Plus_2.0 (Affymetrix) were hybridized with 15 µg amplified RNA, washed with a fluidics station 450 (Affymetrix), and scanned with GeneChip scanner 3000 (Affymetrix). The complete microarray data and MIAME compliant information have been deposited in NCBIs Gene Expression Omnibus (GEO, http://www.ncbi.nlm.nih.gov/geo/) and are accessible through GEO Series accession number GSE12274.
2. *MSC-replicative senescence*: for a direct comparison with age-induced gene expression changes we have reanalyzed our recently published data on replicative senescence in MSC as described below [16]. Here, we have only considered the 9 hybridizations of the different passages of a 44 year old donor (donor 1; passages 2, 3, 4, 5, 6, 7, 8, 10 and 11; passage 9 could repeatedly not be amplified). Two µg of total RNA were amplified, quality controlled and hybridized to the U133_Plus_2.0 Affymetrix chip as described above. The complete microarray data are accessible through GEO Series accession number GSE9593.
3. *HPC-donor age*: CD34^+^ cells were isolated from 4 cord blood samples and from 15 samples of mobilized peripheral blood from donors ranging from 27 to 73 years. 100 ng of total RNA were amplified with the two-cycle target labeling kit (Affymetrix) and 10 µg amplified RNA were hybridized on the GeneChip Human Genome U133_Plus_2.0 platform (Affymetrix). The complete microarray data is accessible through GEO series accession number GSE12277.

### Analysis of microarray data

The three datasets (*MSC-donor age*; *MSC-replicative senescence* and *HPC-donor age*) were independently normalized and analyzed using the same methods: Raw data were quantil normalized [71]. The signal intensities for each gene were then median-normalized and log2 transformed. For subsequent analysis we have only considered probe sets that were detected as present in more than 50% of the hybridisations of each data set. To identify differential gene expression that correlated with increasing age (or passage) we have performed Pavlidis template matching (PTM) using the MultiExperiment Viewer (MeV, TM4) [72]. The template was specified according to the increasing age (or passage) of the different samples. This method ranks genes by statistical score, permitting further analysis to focus on the most significant without the need of discriminatory classification according to age groups [73]. The threshold criterion for matching can be either the magnitude of the correlation coefficient (*R*), or the significance (*P*-value) of the correlation coefficient. Genes that were either up-regulated or down-regulated with a *P*-value <0.01 were further classified by GeneOntology analysis using GoMiner software (http://discover.nci.nih.gov/gominer/) and representation in functional categories was analyzed by Fischer's Exact p-value test. Chromosomal distribution of differentially regulated genes was analyzed by Chromosomal Co-Localization probability calculator (ChroCoLoc) [74].

### Quantitative real-time PCR analysis

Quantification of mRNA expression for candidate genes was performed by real-time quantitative PCR (QRT-PCR) using the ABI PRISM® 7700HT Sequence Detection System Instrument (Applied Biosystems, Applera Deutschland GmbH, Darmstadt, Germany). Total RNA was reverse transcribed by using the high capacity cDNA reverse transcription kit (Applied Biosystems). Primers were obtained from Biospring (Frankfurt, Germany) (table S5). QRT-PCR reactions were performed with the power SYBR® green PCR master mix in a MicroAmp optical 96-well reaction plate with a ABI PRISM® 7700HT sequence detector (Applied Biosystems) according to the manufacturer's instructions. Gene expression levels were normalized to GAPDH expression, which was used as a housekeeping gene.

### Telomere Repeat Amplification Protocol (TRAP) Assay

Cell lysis of CD34-positive cells and HaCaT cells [75] and telomerase assay were performed using the TRAPeze kit (Intergen Company, Oxford, UK, now sold by Q-Biogene, Heidelberg, Germany) as described previously [31].

### 3D telomeric quantitative Fluorescence in situ hybridization (3D-Telo-Q-FISH) analysis

HPC of different donor age were cytospinned to slides, fixed for 10 min in 3.7% formaldehyde buffered with PBS, washed with PBS for 15 min, rinsed in ddH_2_O and air-dried. Cells were labeled with anti-CD34 (Cymbus Biotechnology LTD) detected by a secondary Alexa488-conjugated anti-mouse antibody (Invitrogen). Fixation, washing and air drying were repeated to stabilize the primary-secondary antibody complex. Thereafter, telomere staining was accomplished with a Cy3-labeled PNA probe (PNA Telomere FISH Kit K5326). Hybridisation, image acquisition, signal quantification and recalculation of arbitary units to telomere length in kb was performed as described previously [30].

## Supporting Information

Figure S1 Immunophenotype of MSC. Representative flow cytometry histograms of MSC from human bone marrow are presented (CD13+, CD29+, CD31−, CD34−, CD44+, CD45−, CD73+, CD90+, CD105+, CD146+/−, CD166+, CD184−). There were no age-associated differences in the immunophenotype of MSC from young or elderly donors.
(1.65 MB TIF)

Figure S2 In vitro differentiation of MSC. MSC of young and elderly donors were simultaneously differentiated along adipogenic or osteogenic line. Fat accumulation was visualized by Oil Red-O staining. Osteogenic differentiation was visualized by Alizarin red staining. There were no age-associated differences in the differentiation potential of MSC. (scale bar = 100 µm)
(5.28 MB TIF)

Figure S3 beta-galactosidase staining of MSC. Senescence associated beta-galactosidase staining increases in the later passages of MSC (scale bar = 100 µm).
(7.44 MB TIF)

Figure S4 RT-PCR analysis of independent donor samples. Age associated gene expression changes in MSC were validated in independent donor samples of young (26 and 30 years old), median (35 and 45 years) and elderly donors (76 and 85 years old). Differential expression was analyzed by RT-PCR (A). Furthermore, we have isolated HPC from two additional cord bloods, three young (20, 26, 26 years old) and three elderly donors (49, 55, 58 years old) (B). Differential gene expression was always calculated in relation to the mean of young samples. The mean fold-ratio (±SD) is demonstrated. RT-PCR results (red) validated age associated gene expression changes as observed in microarray data (blue).
(1.47 MB TIF)

Figure S5 Gene expression changes in MSC upon serial passaging. Differential gene expression of different passages of the same MSC preparation (44 years old) was analyzed by Affymetrix GeneChip technology as described before [16]. This data was now reanalysed by different statistical methods for direct comparison of datasets. Analysis by Pavlidis template matching revealed that 1257 expressed sequence tags (ESTs) were significantly up-regulated (red; P<0.01) and 698 ESTs were down-regulated (green) upon replicative senescence.
(3.78 MB TIF)

Figure S6 Cell numbers in mobilized peripheral blood. The number of MNC per ml blood (A), of CD34+ cells (B) and the percentage of CD34+ HPC in MNC (C) was determined for cord blood samples and for the mobilized peripheral blood samples. There was no correlation between donor age and the number of HPC in the blood.
(0.57 MB TIF)

Figure S7 Functional categories of differentially expressed genes. GeneOnthology analysis was performed for the subsets of genes that were significantly up-regulated (red) or down-regulated (green) in MSC-donor age (A), HPC-donor age (B) and MSC-replicative senescence (C). The number of non-redundant genes in each category was compared to all genes (grey) on the microarray. The 10 most significant categories are depicted and the percentages of genes that contributed to representative categories are presented (P<0.0001).
(5.09 MB TIF)

Figure S8 Serial dilution for the TRAP assay. All cell lysates were diluted with extract from telomerase-negative fibroblasts in order to maintain comparable protein concentrations in the extract. All lysates show a dilution-dependent reduction in telomerase activity, demonstrating that reduced activity in some samples was not due to the presence of inhibitors. H+ = HaCaT lysate with RNase, LB = lysis buffer control, IC = internal 34 bp standard
(5.02 MB TIF)

Table S1 Gene list of differentially expressed genes in aging of MSC. This table summarizes significantly age-induced (red; 99 ESTs) and age-repressed genes (green; 85 ESTs). The table contains the following columns: 1) Affymetrix ID, 2) Gene Titel, 3) Gene Symbol, 4) Chromosomal location, 5) number of present detections in 12 hybridizations, 6) Correlation coefficient with the template (PTM-matching), and 7) P-value.
(0.06 MB XLS)

Table S2 Gene list of differentially expressed genes in replicative senescence of MSC. This table summarizes significantly senescence-induced (red; 1257 ESTs) and senescence-repressed genes (green; 698 ESTs). The table contains the following columns: 1) Affymetrix ID, 2) Gene Titel, 3) Gene Symbol, 4) Chromosomal location, 5) number of present detections in 9 hybridizations, 6) Correlation coefficient with the template (PTM-matching), and 7) P-value.
(0.40 MB XLS)

Table S3 Gene list of differentially expressed genes in aging of HPC. This table summarizes significantly age-induced (red; 776 ESTs) and age-repressed genes (green; 704 ESTs). The table contains the following columns: 1) Affymetrix ID, 2) Gene Titel, 3) Gene Symbol, 4) Chromosomal location, 5) number of present detections in 19 hybridizations, 6) Correlation coefficient with the template (PTM-matching), and 7) P-value.
(0.31 MB XLS)

Table S4 Differentially expressed genes are over-represented in chromosomal bands.
(0.02 MB XLS)

Table S5 Primer list.
(0.02 MB XLS)
