# Supplementary figures and images for: Aging and Replicative Senescence Have Related Effects on Human Stem and Progenitor Cells
Source: PLoS One. 2009 Jun 9;4(6):e5846. doi: 10.1371/journal.pone.0005846 (PMC2688074; doi:10.1371/journal.pone.0005846)

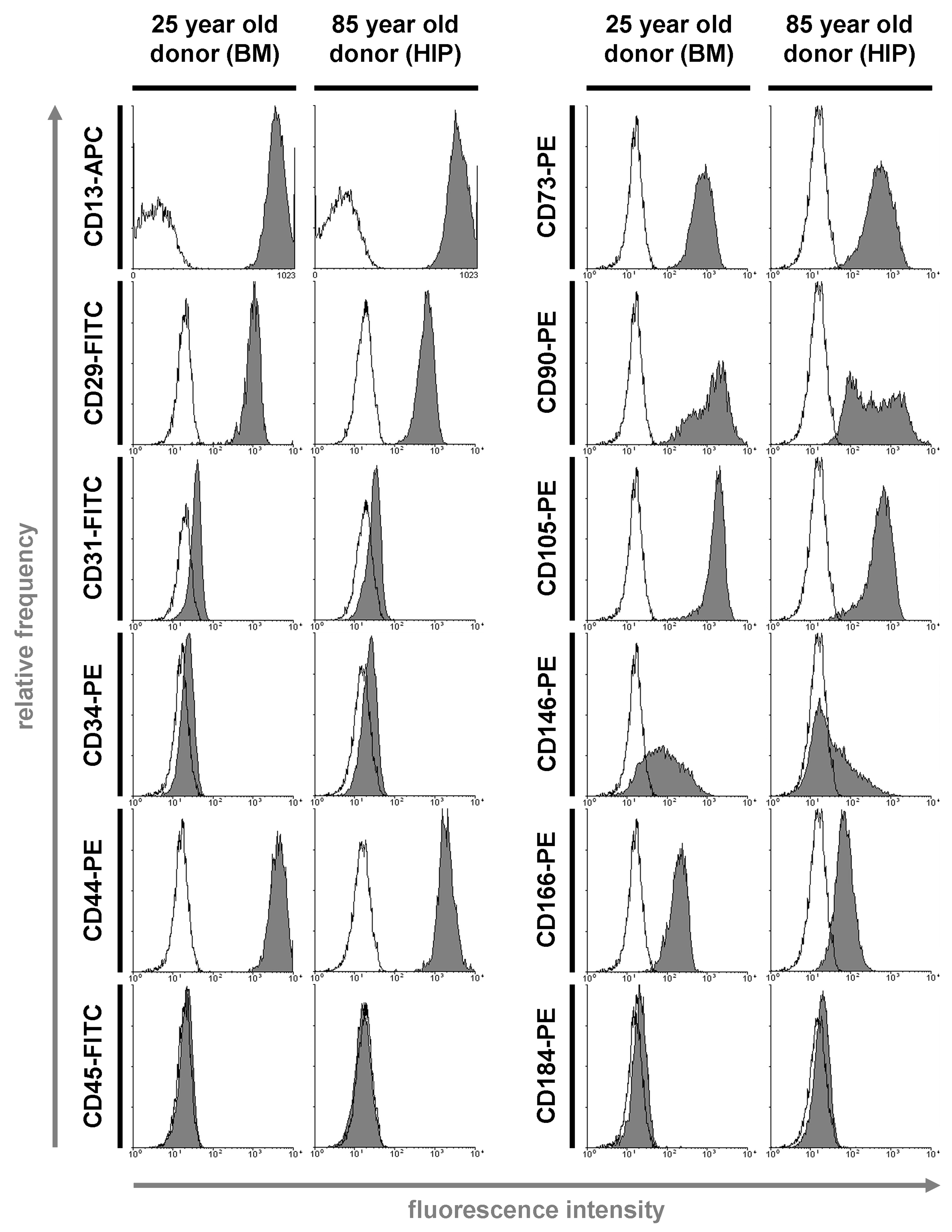

Supplement: Figure S1 — Immunophenotype of MSC. Representative flow cytometry histograms of MSC from human bone marrow are presented (CD13+, CD29+, CD31−, CD34−, CD44+, CD45−, CD73+, CD90+, CD105+, CD146+/−, CD166+, CD184−). There were no age-associated differences in the immunophenotype of MSC from young or elderly donors. (1.65 MB TIF) [file pone.0005846.s001.tif]

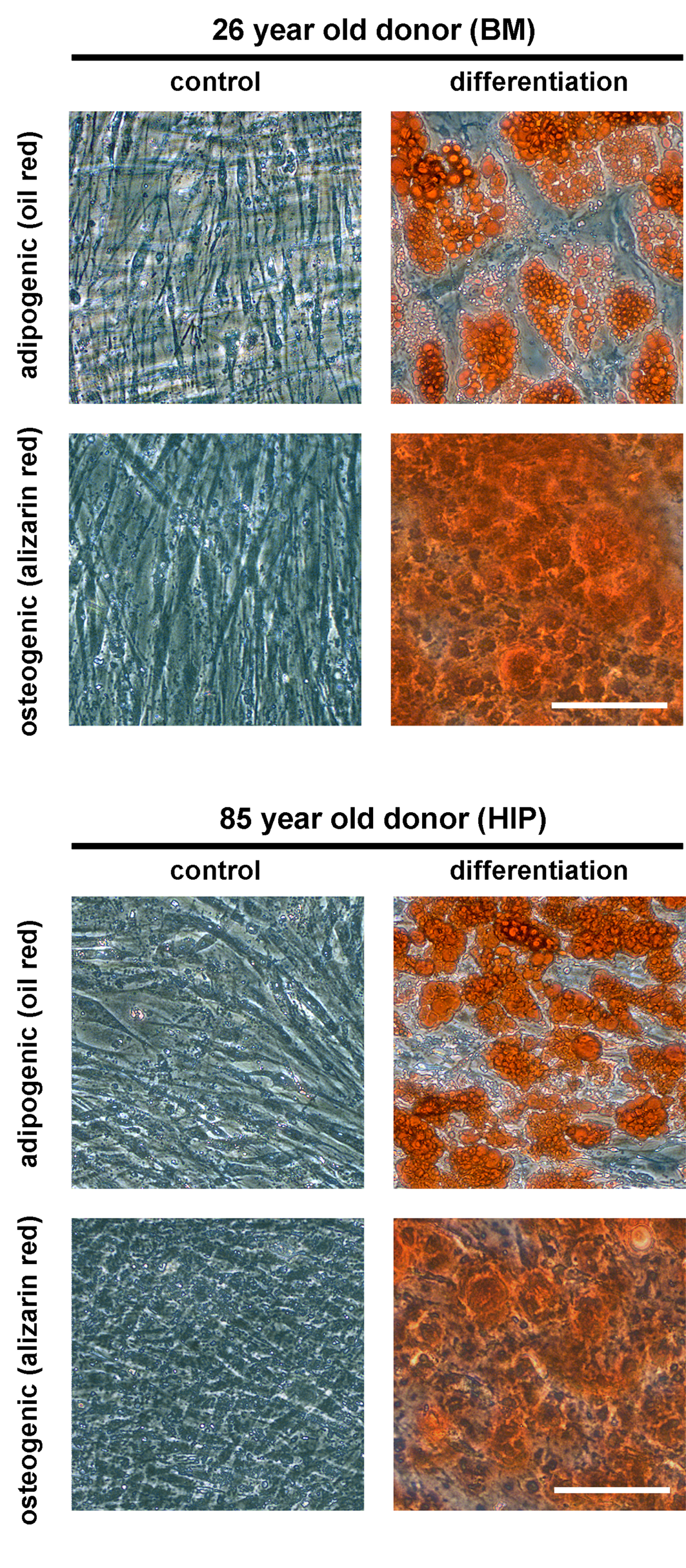

Supplement: Figure S2 — In vitro differentiation of MSC. MSC of young and elderly donors were simultaneously differentiated along adipogenic or osteogenic line. Fat accumulation was visualized by Oil Red-O staining. Osteogenic differentiation was visualized by Alizarin red staining. There were no age-associated differences in the differentiation potential of MSC. (scale bar = 100 µm) (5.28 MB TIF) [file pone.0005846.s002.tif]

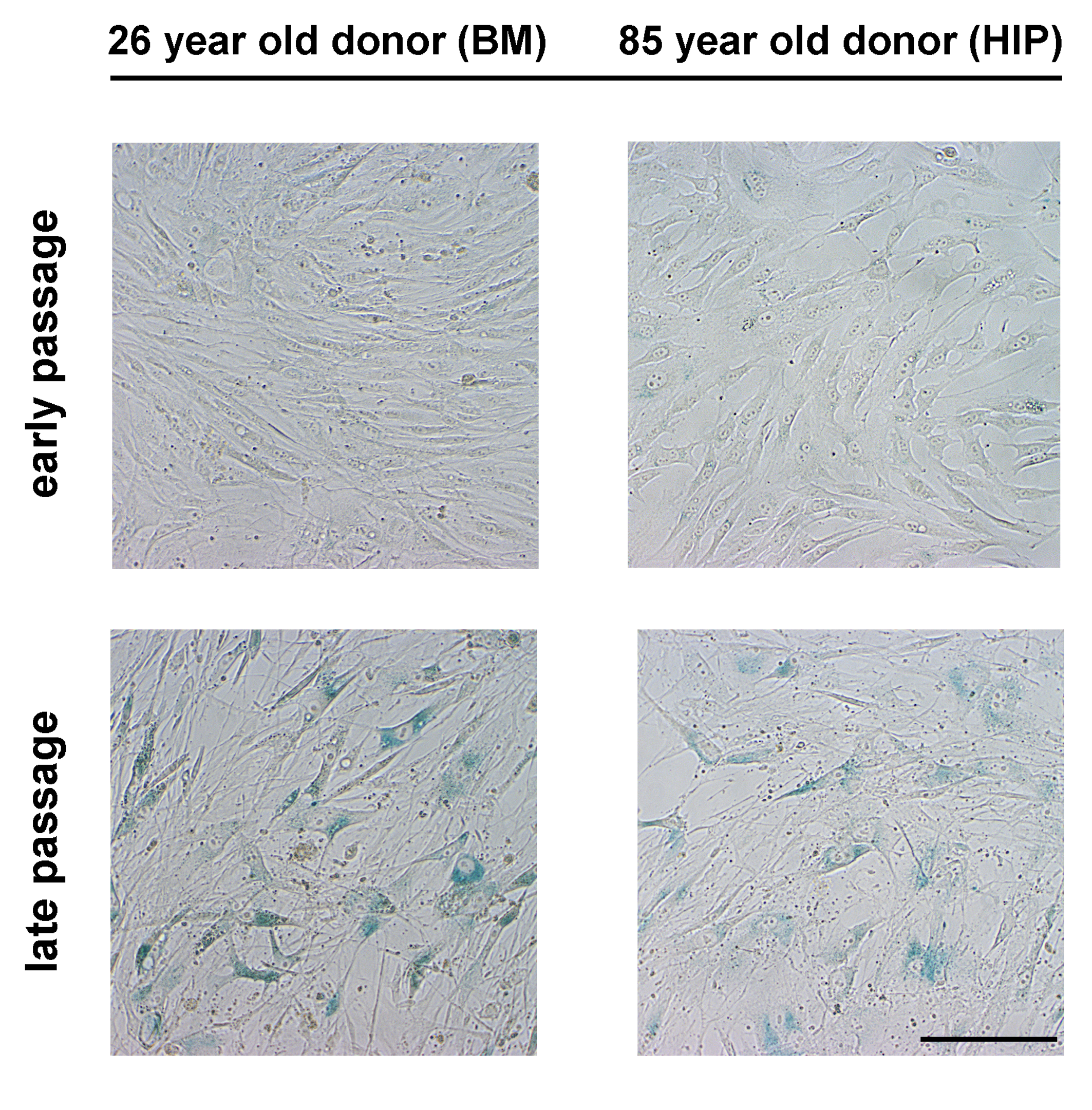

Supplement: Figure S3 — beta-galactosidase staining of MSC. Senescence associated beta-galactosidase staining increases in the later passages of MSC (scale bar = 100 µm). (7.44 MB TIF) [file pone.0005846.s003.tif]

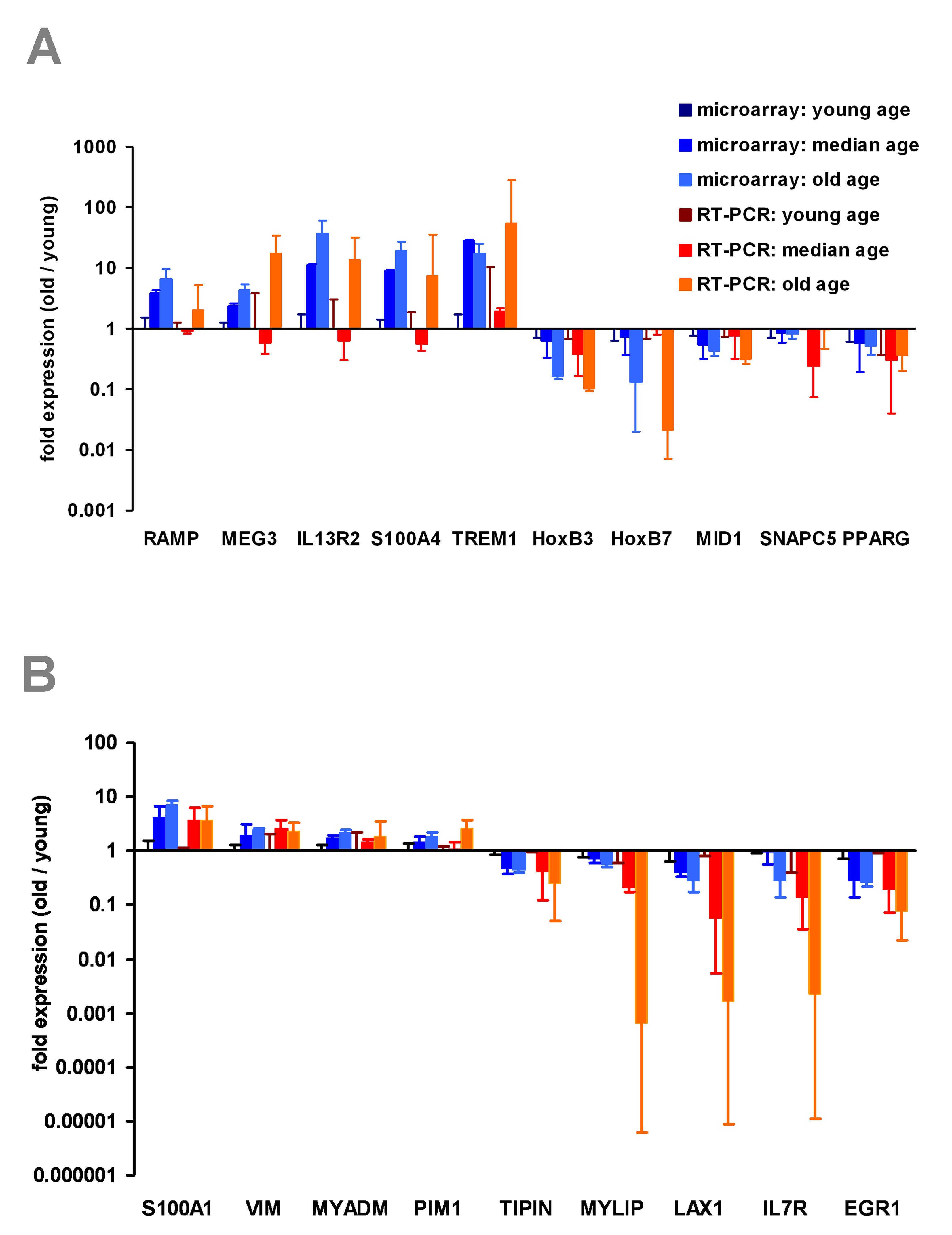

Supplement: Figure S4 — RT-PCR analysis of independent donor samples. Age associated gene expression changes in MSC were validated in independent donor samples of young (26 and 30 years old), median (35 and 45 years) and elderly donors (76 and 85 years old). Differential expression was analyzed by RT-PCR (A). Furthermore, we have isolated HPC from two additional cord bloods, three young (20, 26, 26 years old) and three elderly donors (49, 55, 58 years old) (B). Differential gene expression was always calculated in relation to the mean of young samples. The mean fold-ratio (±SD) is demonstrated. RT-PCR results (red) validated age associated gene expression changes as observed in microarray data (blue). (1.47 MB TIF) [file pone.0005846.s004.tif]

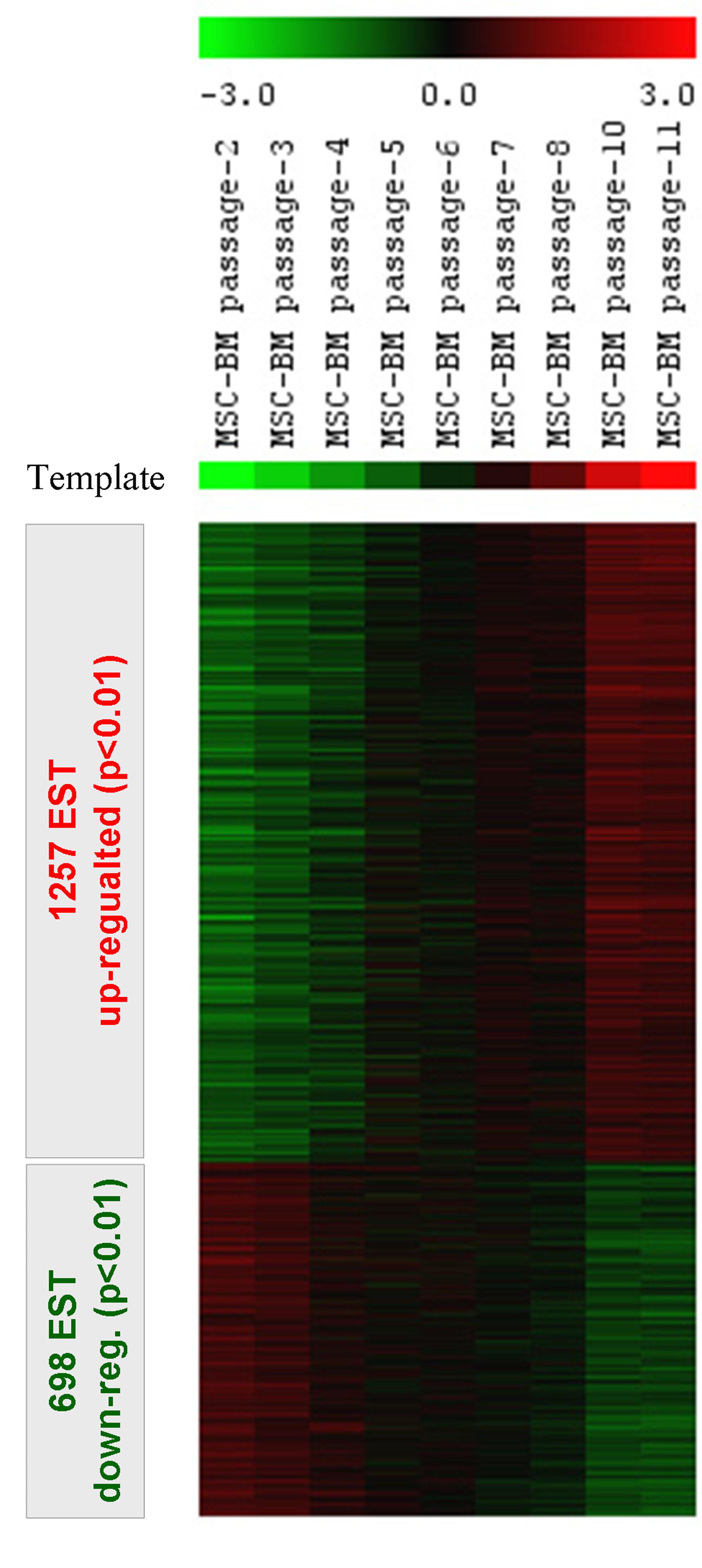

Supplement: Figure S5 — Gene expression changes in MSC upon serial passaging. Differential gene expression of different passages of the same MSC preparation (44 years old) was analyzed by Affymetrix GeneChip technology as described before [16]. This data was now reanalysed by different statistical methods for direct comparison of datasets. Analysis by Pavlidis template matching revealed that 1257 expressed sequence tags (ESTs) were significantly up-regulated (red; P<0.01) and 698 ESTs were down-regulated (green) upon replicative senescence. (3.78 MB TIF) [file pone.0005846.s005.tif]

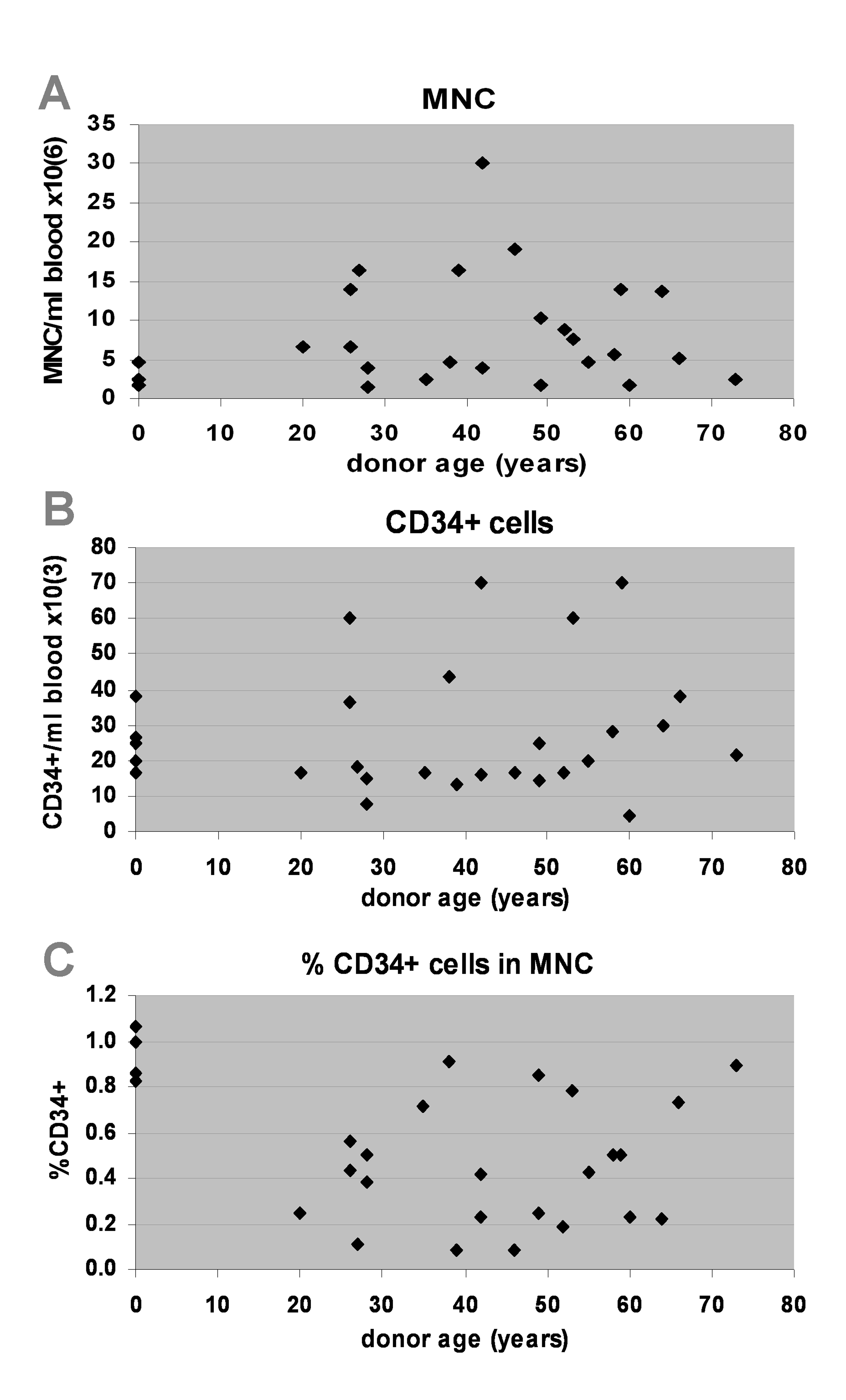

Supplement: Figure S6 — Cell numbers in mobilized peripheral blood. The number of MNC per ml blood (A), of CD34+ cells (B) and the percentage of CD34+ HPC in MNC (C) was determined for cord blood samples and for the mobilized peripheral blood samples. There was no correlation between donor age and the number of HPC in the blood. (0.57 MB TIF) [file pone.0005846.s006.tif]

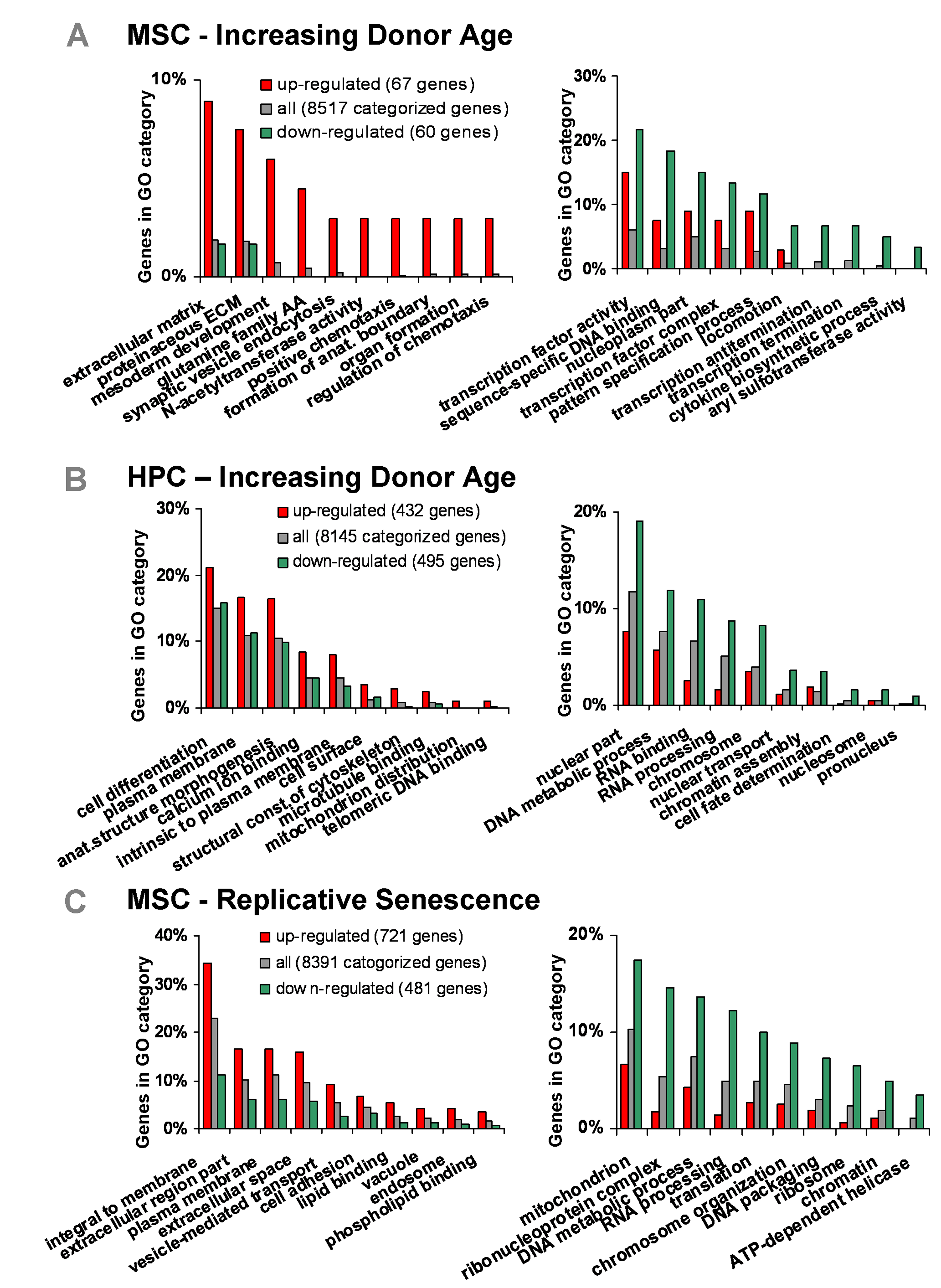

Supplement: Figure S7 — Functional categories of differentially expressed genes. GeneOnthology analysis was performed for the subsets of genes that were significantly up-regulated (red) or down-regulated (green) in MSC-donor age (A), HPC-donor age (B) and MSC-replicative senescence (C). The number of non-redundant genes in each category was compared to all genes (grey) on the microarray. The 10 most significant categories are depicted and the percentages of genes that contributed to representative categories are presented (P<0.0001). (5.09 MB TIF) [file pone.0005846.s007.tif]

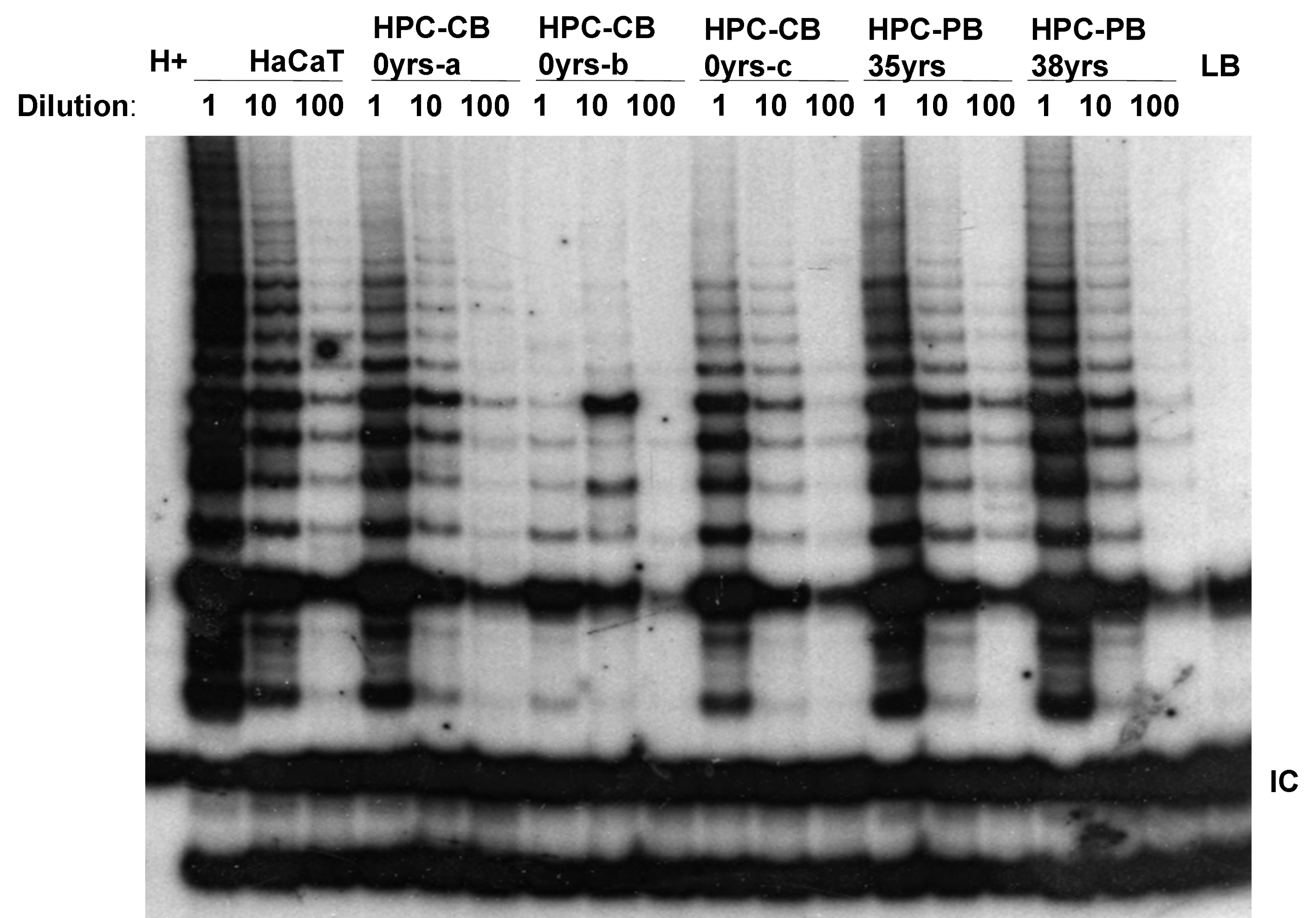

Supplement: Figure S8 — Serial dilution for the TRAP assay. All cell lysates were diluted with extract from telomerase-negative fibroblasts in order to maintain comparable protein concentrations in the extract. All lysates show a dilution-dependent reduction in telomerase activity, demonstrating that reduced activity in some samples was not due to the presence of inhibitors. H+ = HaCaT lysate with RNase, LB = lysis buffer control, IC = internal 34 bp standard (5.02 MB TIF) [file pone.0005846.s008.tif]
